# High sensitive space electric field sensing based on micro fiber interferometer with field force driven gold nanofilm

**DOI:** 10.1038/srep15802

**Published:** 2015-10-28

**Authors:** Tao Zhu, Liming Zhou, Min Liu, Jingdong Zhang, Leilei Shi

**Affiliations:** 1 Key Laboratory of Optoelectronic Technology and Systems (Ministry of Education), Chongqing University, Chongqing 400044, China

## Abstract

The traditional electrical field sensing can be realized by utilizing electro-optic materials or liquid crystals, and has limitations of easy breakdown, free assembly and difficult measurement of low-frequency. Here, we propose a new method to realize safe measurement of spatial dynamic electric field by using a micro fiber interferometer integrated with gold nanofilm. The energy of the electric charge received through antenna forms the intrinsic electric field with two micro electrodes, one of which is the 120 nm gold film vibration beam micromachined by femtosecond lasers and integrated with the micro fiber. The change of the intrinsic electric field force due to the spatial electric field will cause the vibration of the film beam. By demodulating the output signal of the micro fiber interferometer, the electric field can be measured. We demonstrate the detectable frequency ranges from tens of Hz to tens of KHz, and the minimum electric field intensity is ~200 V/m at 1 KHz. Our electric field measurement technology combining optical fiber interference with gold nanostructures shows the advantages of security, high sensitivity, compact size, and multiplexed multi-point and remote detection.

Electric field sensing is important for the prevention of electromagnetic interference^1^, voltage balancing^2^,^3^,^4^, the shielding of near-field electromagnetic radiation^5^ and other special applications such as detecting charges^6^, electrostatic precipitation^7^ and millimeter-wave to lightwave signal converter^8^. Although traditional electric field sensors, such as steered-electron^9^ (detectable range is from several mV/m to dozens of V/m), spherical electric field probe^10^ (<12 kV/m), bistable microelectronic circuit^11^, and THEMIS three-axis electric field instrument^12^ (mV/m to several V/m), can work precisely in some applications, they can be easily damaged along with their subsequent circuits owing to unpredictable high intensity of electric field and needs active devices which makes them unsuitable for remote detection. Also, the metallic circuits and signal transmission cables are susceptible to the electromagnetic interference.

In recent years, the optical electric field detection^6^,^13^,^14^,^15^,^16^,^17^,^18^,^19^,^20^,^21^ has attracted increasing attentions. They possessed good qualities such as remote and safety measurement, passive component, integrated structure, easy networking based on WDM technology, extremely weak interfering with environment and electric field source. The optical electric field measurements in time domain are based on two kinds of materials^14^,^15^,^16^,^17^,^18^,^19^,^20^,^21^, respectively. The first one is electro-optic (EO) materials^14^,^15^,^16^,^17^,^18^,^19^ which is used for MHz to GHz RF frequency electric field sensing primarily. The corresponding detectable ranges are above 2.5 V/m (or several mW/m^2^ of the minimum detectable electromagnetic energy flux density) for ref. 14, and 19 V/m to 23 kV/m for ref. 15, But it has been rarely reported in the low frequency applications below dozens of kilohertz because of the irregular frequency response caused by the piezoelectric effect of the EO^22^,^23^,^24^,^25^ materials or other effects^15^,^26^. The second material is liquid crystal^16^,^18^,^20^,^21^ which is applicable for the measurement of electric field in low frequency, and the reported detectable ranges of electric field intensity are above dozens of KV/m for ref. 20 and 1 to 4.1 kV/mm for ref. 21.

Considering that most low frequency electric field sensing are employed in the electric power system, it’s very important to realize safe measurement of low-frequency electric field with high sensitivity. For the first time, we propose a new method to make a sensor by integrating the antenna and the optical fiber whose detectable frequency ranges from tens of Hz to tens of KHz. The minimum detectable electric field intensity is ~200 V/m at 1 KHz, and the maximum is about 5 kV/m according to the half-wave electric field intensity. The sensitivity can be further improved by modifying the structure and the parameters of the antenna (the limit of minimum detectable electrical field intensity can be as low as ~0.015 V/m with the length of the antenna of ~27 mm). The key component of the sensor is the micro fiber interferometer integrated with gold nanofilm. The sensing is achieved through the micro area in the sensor with strong electric field formed by the coupling between the antenna and the space electric field. The gold nanofilm can function as the electrode of the micro area, meanwhile, it deforms under the ultra-weak electrostatic force caused by the strong electric field. The vibration beam formed by the gold nanofilm can be the reflector of the Fabry-Perot (F-P) interferometer. Hence, the change of the space electric field can be detected through demodulating the change of cavity length of the interferometer under the electrostatic force.

## Results Section

### Experimental setup

**Figure 1:**
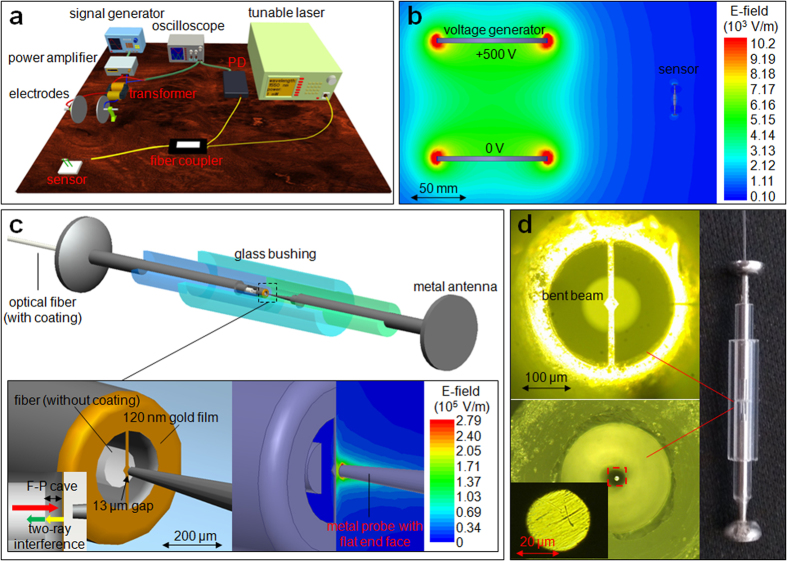
The schematics of electric field sensing. (**a**) Experimental setup. (**b**) The simulated distribution of electric field intensity of the sensing system. (**c**) The structure and inner electric field intensity of the sensor. (**d**) Photos of the sensor.

The measurement system to detect the near space electric field is shown in Fig. 1. Light signal from the tunable laser AQ4321D with output wavelength of 1520–1620 nm and power of 0.1~4 mW passes through a coupler (50:50) and reaches to the sensor. The change of the space electric field is modulated to the signal light through the sensor. And the signal light passes through the same coupler to the photodetector (PDA20C/M, PD) reversely. The output from the PD are imported into the subsequent signal processing equipments, such as the frequency transformer, the amplifier and the oscilloscope to record and demodulate the change of the space electric field.

### The sensor structure

The structure of the sensor is shown in Fig. 1c. The sensor is composed of two parts: The first part is the built-in plate capacitor constructed by the 120 nm gold nanofilm vibration beam and the end surface of the metal probe. The metal rods connected to the gold nanofilm and the metal probe, respectively, forms the space electric field receiving antenna. The built-in electric field of the plate capacitor is equivalent to the amplification results of the space electric field of the antenna because of electrostatic induction effect. The second part is the F-P interferometer formed by the other side of the gold nanofilm vibration beam and the end surface of the optical fiber. The change of the built-in electric field of the plate capacitor has the ability of driving the vibration of the beam so as to realize the modulation of the interference signal of the F-P interferometer.

**Figure 2:**
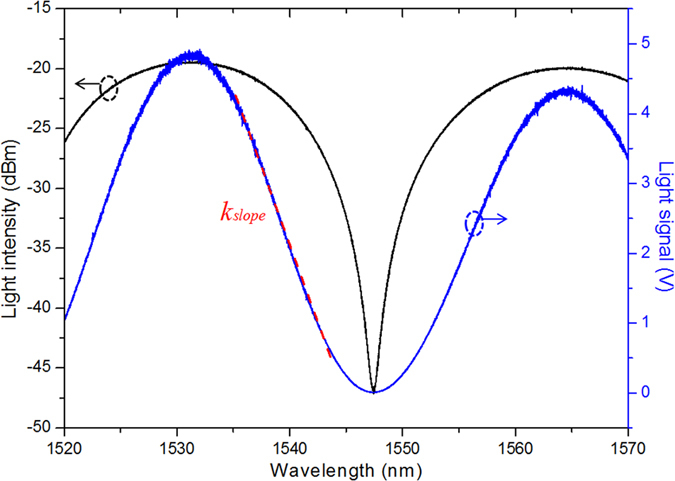
The reflection spectrum of the sensor. It is measured with spectrometer (Si720).

For the exploration of suitable operating wavelength, the reflection spectrum of the F-P interferometer is measured and shown in Fig. 2. We can see that the light signal with the amplitude of PD output voltage from 3.9 V to 0.7 V has a linear relationship with the wavelength from 1536 nm to 1543 nm with slope *K*_*slope*_. The operating wavelength is determined as 1540.5 nm and the corresponding amplitude of output voltage is 2.0 V.

### The principle of the sensor

**Figure 3:**
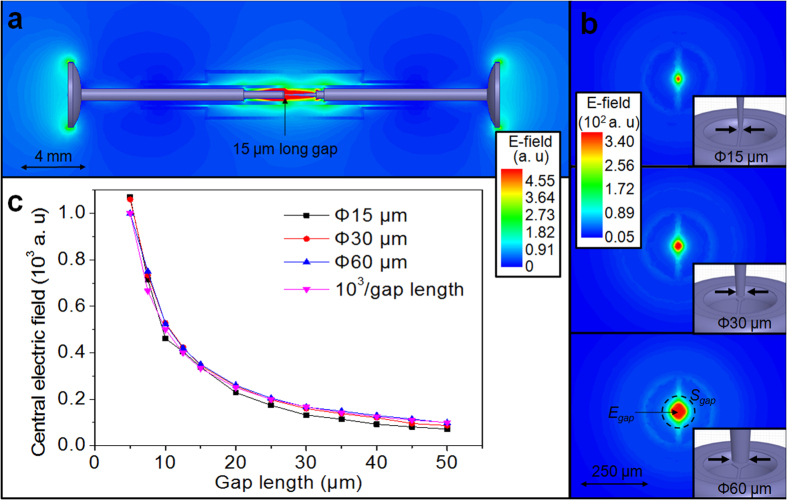
The simulated distribution of electric field intensity within the sensor. (**a**) Distribution within the whole sensor. (**b**) The distribution in the middle plane between vibration beam and probe surface. (**c**) The relationship between the central electric field intensity of the gap and the gap length.

It should be noted that only the near-field low-frequency electric field distribution is considered in the absence of magnetic field. To quantitatively describe the sensor’s characteristics of electric field sensing, we have simulated the induced electric field intensity with different structural parameters (see Fig. 3). It can be seen that although the electric field intensity in the gap is large, the electric field exists in a large scale of area, which is unexpected and unavoidable (see Fig. 3a). The central electric field intensity of the gap remains almost unchanged when we tune the sizes of probe end face and central vibration beam. These can be explained that the induced quantity of electric charge gathered in the gap is only a very small part of the whole quantity on a much larger scale of area. In other words, the equivalent capacitance within the sensor almost keeps unchanged even the area of end face and central vibration beam are modified. It can be concluded that the electric field intensity of the gap *E*_*gap*_ is independent of the gap area *S*_*gap*_, but is proportional to the amplitude of external spatial electric field intensity *E*_*ext*_. The amplitude of electrostatic force is proportional to the product of *E*_*gap*_ and *S*_*gap*_. Besides, *E*_*gap*_ is almost inversely proportional to the gap length *L*_*gap*_ according to Fig. 3c. We can derive the relationship between the peak light signal change Δ*I*_*peak*_ and the peak external space electric field intensity *E*_*extpeak*_ (parallel to the direction of the metal rods) as equation (1) and (2) (see more details in Supplementary information):


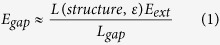



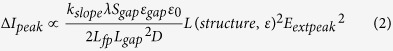


where *ε*_*gap*_ is the relative inductivity of gap (here it is 1), *ε*_*0*_ is the permittivity of free space, *L*(*structure*, *ε*) is the equivalent antenna length that depends on the structure of antenna (except the gap length) and the insulated packaging material as well as their relative inductivity *ε* and gap *ε*_*gap*_. The optimal *L*(*structure*, *ε*) approximate to the total length of two sections of antenna, *D* is the stiffness factor of the vibration beam, and *L*_*fp*_ is the cavity length of F-P (here it is 37.4 μm) interferometer.

### Electric field test

**Figure 4:**
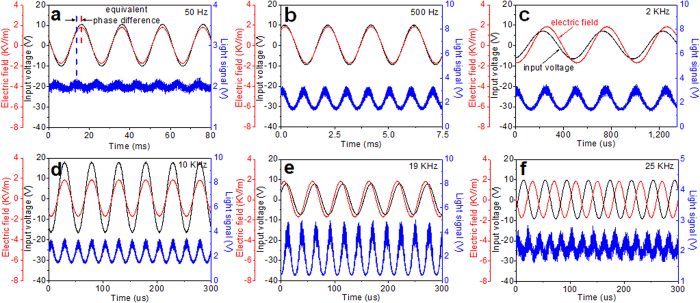
The waveforms of electric field sensing including the input voltages of transformer (black), electric field intensities around the sensor (red) and light signals (blue). (**a**) 50 Hz. (**b**) 500 Hz. (**c**) 2 KHz. (**d**) 10 KHz. (**e**) 19 KHz. (**f**) 25 KHz.

**Figure 5:**
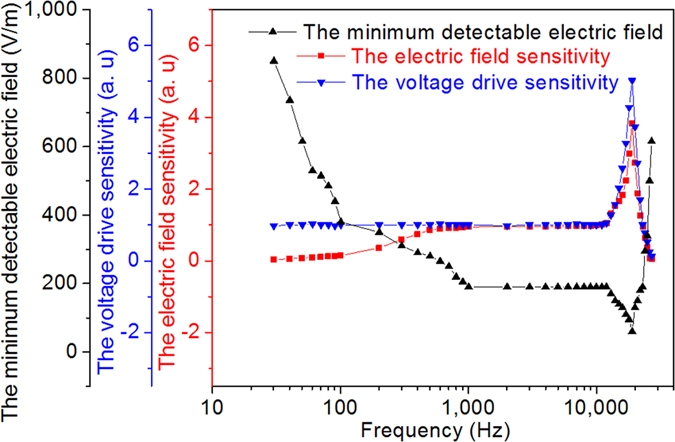
The frequency characteristics of the minimum detectable electric field intensity, the voltage drive sensitivity, and the electric field sensitivity of our sensor.

The low-frequency electric field experiment is carried out when the frequency ranges from 30 Hz to 27 KHz with maximum electric field intensity of 3600 Vpp/m, and the experimental results are shown in Fig. 4. As for the frequency below ~500 Hz, a response decline (see Fig. 5) along with an equivalent phase difference (see Fig. 4a) exists. There is a response peak at 19 KHz. The minimum detectable electric field intensity is ~200 V/m at 1 KHz which is measured by decreasing the electric field intensity until the optical signal cannot be extracted from the background noise. In our measuring system the test signal generator of 10 V output is used to directly transmit the signal to the antenna poles through wires in order to replace the space electric field source used before. The dimensionless values of voltage drive sensitivity shown in Fig. 5 are the normalized serial values of light signal change obtained when a sweep frequency voltage of same amplitude was applied on the sensor. The result indicates that the sensor has flat response (with very small equivalent phase difference) below 13 KHz and the peak also exists at 19 KHz (with large equivalent phase difference).

### Discussion Section

The response of our sensor is flat at intermediate frequency of 500 Hz~13 KHz and weak at low frequency around 0 Hz. The obvious response peak occurs at high frequency of 19 KHz, which is understandable in that the inherent resonant frequency of the vibration beam could be ~38 KHz (it is also expected to be the maximum modulation frequency of the F-P interferometer for our sensor). We can explain the decrease of the response at low frequency around 0 Hz from the point of impedance. When the resistance of the glass bushing of the sensor is assumed to be infinite, the sensor has good amplitude-frequency response around 0 Hz. However, in reality, the resistance of the glass bushing is finite, and it’s even smaller than the capacitive reactance of the sensor, which results in the smaller impedance of the sensor considering the parallel connection of the resistance and capacitance. Hence, around 0 Hz, the strong insulating external air occupies much more voltage than give it to the sensor if we considering them along the electrodes of electric field source in a voltage dividing model. The frequency response around 0 Hz can be flat by using the packaging material with higher resistivity. We also can understand the response of the sensor from the viewpoint of the phase relationship. It is the parallel connection of the resistance and capacitance that leads to the high-pass filtering effect of the sensor, i.e., compared with *E*_*ext*_, the phase of *E*_*gap*_ has a phase lead, whereas Δ*I* has a phase delay due to the mechanical characteristics of the gold film vibration beam. These two opposite effects cause the light intensity change has almost the same phase with the electric field intensity at intermediate frequency, however, has a phase lead at low frequency and a phase delay at high frequency (see Supplementary Figure S2). By treating the vibration beam as a spring oscillator, the light intensity change can be described as:


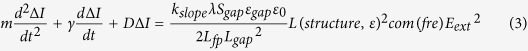


where *m* and *γ* are the equivalent mass and damping coefficient of the vibration beam respectively. *com(fre)* is the function to compensate the amplitude and frequency simultaneously. When electric field generating source is assured, *com(fre)* can be calculated through Fig. 5 after *m* and *γ* are determined. In actual applications, since period instead of DC signal is what we attempt to measure, there is no need to use the non-modulated light signal which carried notable noise as the zero electric field reference point. Instead, according to the square relation between Δ*I* and *E*_*ext*_, we can use a medium intensity electric field and the sensor’s corresponding output value of light signal change for calibration, to find out the intensity of target electric field.

Based on the parameters of the sensor, the upper limit of the frequency response is ~20 KHz. The faster charge and discharge rate compared with the deformation velocity of the beam, which is implied by the existence of peak response at high frequency, indicates that upper limit of the frequency can be further increased by reducing the mass of the center of vibration beam and the cavity air resistance (vacuumization) or the designing of higher stiffness factor connection of golden film. The minimum detectable electrical field intensity is ~200 V/m (with the length of internal gap of 13 μm) in the frequency range between 1 KHz to 12 KHz, which will be bigger at lower frequency but smaller at higher frequency. It can be improved by enlarging the external size of the antenna pole, especially the length, or by reducing the length of gap within the sensor. It can be estimated that the minimum detectable deflection of vibration beam is ~1 nm, according to the light intensity noise of the interferometer in the sensor. If the length of internal gap is decreased to 1 nm, whereas the size of the antenna is kept unchanged, with Equation (1) we can calculate that the minimum detectable electrical field intensity can be as low as ~0.015 V/m (with the total length of the antenna of ~27 mm and the equivalent length of ~13 mm). As for the theoretical sensitivity, our sensor is superior to other optical sensors requiring the gap length of inner electrode be greater than 100 nm for waveguide. Above analysis also indicates that our sensor could be further minimized with nanotechnology to smaller size. As for the low-frequency electric field response, the mixed metal impurity material is applied in the electro-optic material electric field sensor for optical waveguide fabrication and the high frequency sensitivity is improved by sacrificing the low-frequency electric field response which is depending on the insulativity of the sensor. And the liquid crystal electric field sensor cannot improve the low frequency sensitivity by increasing the resistance of the liquid crystal. Comparatively, there exists an air gap in our sensor, which has the capability of producing very low frequency response with the help of high insulation resistivity. The frequency response around 0 Hz can be flatter by using the packaging materials with higher resistivity. Also, it’s easier to realize the package of the sensor with the antenna and the optical fiber. There is no doubt that our sensor is competitive due to the excellent performance.

Additionally, the electrostatic force generated on the vibration beam ranges from 10^–11^ to 10^–9^ N according to the internal electric field intensity (see Supplementary equation S(2)). The maximum calculated deformation (the central deflection) can be up to a few hundred nanometers based on the actual measurement of the optical signal amplitude. However, the large electric field can cause the large deformation of the vibration beam, leading to the distortion of the optical signal because of the perturbation of *E*_*gap*_ caused by the change of *L*_*gap*_. The vibration beam can be affected by the biasing force due to the square field effect, which will have the effect of changing the operating wavelength of the sensor (see Supplementary Figure S3), and correspondingly, the dynamic range of the sensor. In actual applications, fastening the mechanical structure of the sensor, especially the F-P cavity and the gap, is helpful to eliminate the fluctuation of original light intensity and electric field intensity response. The effects of temperature and vibration on the electric field measurement should also be considered. It should be suggested that our sensor be fixed in stable platforms, and the temperature compensation structure and algorithm be introduced to overcome the structure deformation induced by temperature variation. The power consumption consisted of the very small amount of energy extracted from E-field source and lasers source power (0.1~4 mW), which depends on the initial charge energy of sensor’s equivalent capacitance (<0.1 μJ), the initial kinetic energy of gold film (<10^−22^*f*^ 2^ J/s^2^, *f* is the electric field frequency), air resistance power consumption (<1 nW), and sensor resistance heat (from resistance of packaged glass, <10^−11^ W). With the property of miniaturization and small cross affection with electric field source or environment, our sensor is suitable for directional space electric field sensing or even three-dimensional sensing if three sensors are assembled in different orientations.

In summary, we have demonstrated the feasibility of a new method to realize safe measurement of spatial dynamic electric field by using a micro fiber interferometer integrated with gold nanofilm. Although the equivalent driven internal electric field intensity is in the same class as existed optical sensing mechanisms, the external to internal electric field gain factor of our sensor could be bigger than any other optical sensors through the narrowing of the gap length towards 1 nanometer scale without principle constraints (which existed EO^14^,^15^ methods require more than 300 nm gap for optical waveguide, and the LC^20^ method requires 30 μm). Which means the limiting minimum detectable intensity of ours (15 mV/m) is better than other optical sensors^14^,^15^,^20^,^21^ if with same structural size and in low frequency region. And the electrical resistivity of our sensor (air dielectric) could be much bigger than any others (EO material or LC dielectric), which illustrates the competitiveness of our sensor in low frequency electric field detection such as optical electric pulse detection^19^,^27^, power grid monitoring and the orientation measurement of electric field.

## Methods Section

### Electric field generation

Electrical signal of dozens Hz to KHz from signal generator is inputted into a power amplifier and then act as the input of the transformer (there are several different transformers used for different sections of frequency). The actual transformer ratio and phase characters have been pre-measured to figure out the high voltage output of transformer. The high voltage is used to generate the required medium to high intensity electric field. The electric field intensity around the sensor is calculated from the simulation of the electric field distribution.

### Fabrication of golden vibration beam

The end of stainless steel capillary (internal diameter 250 μm) is covered with a whole piece of gold film. It is fabricated by a femtosecond laser with high-precision processing platform. The central wavelength of femtosecond laser is about 790 nm. The repeat frequency rate of pulse is 1 KHz. And the focused spot size is ~3 μm. The energy of laser pulse is adjusted to be just enough for gasifying the gold (~1 μJ) to ensure the smooth edge of beam.

## Additional Information

**How to cite this article**: Zhu, T. *et al.* High sensitive space electric field sensing based on micro fiber interferometer with field force driven gold nanofilm. *Sci. Rep.* **5**, 15802; doi: 10.1038/srep15802 (2015).

## Supplementary Material

Supplementary Information
