# Supplementary material for: High sensitive space electric field sensing based on micro fiber interferometer with field force driven gold nanofilm
Source: Sci Rep. 2015 Oct 28;5:15802. doi: 10.1038/srep15802 (PMC4623471; doi:10.1038/srep15802)
Supplement: Supplementary Information [file srep15802-s1.doc]

Supplementary Information

High sensitive space electric field sensing based on micro fiber interferometer with field force driven gold nanofilm

Tao Zhu, Liming Zhou, Min Liu, Jingdong Zhang & Leilei Shi

Key Laboratory of Optoelectronic Technology and Systems (Ministry of Education), Chongqing University, Chongqing 400044, China.


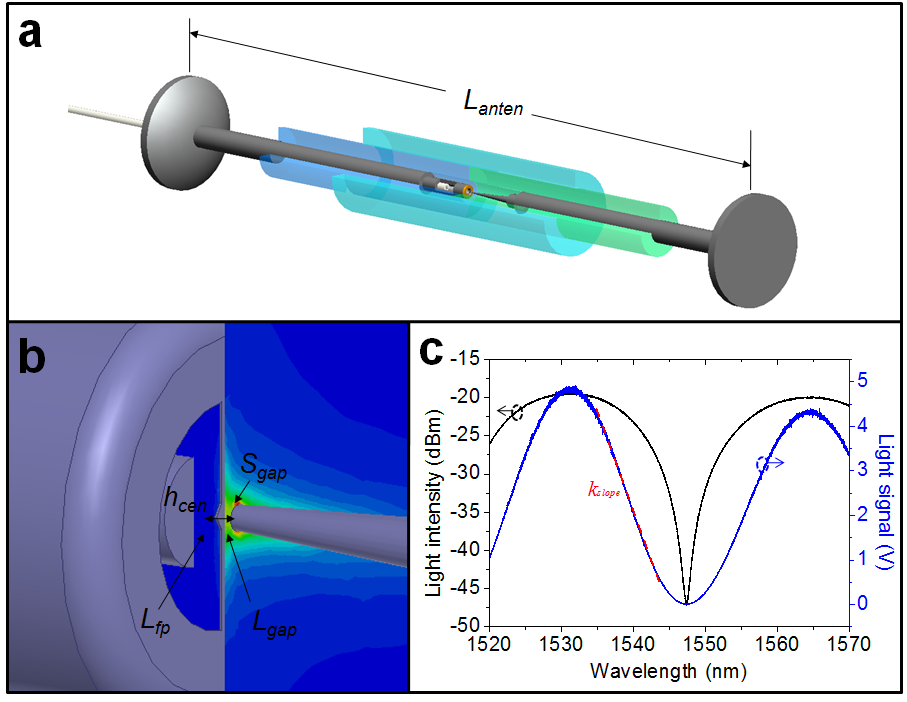
**Figure S1 | The structure of the sensor for the illustration of principle.** (a) and (b) are the structure and inner structure of the sensor, respectively. (c) The reflection spectrum of the sensor.

According to the electric field distribution of electrostatic induction, the inner voltage of gap *V_gap_* is less than or near less than *E_ex_*_t_*L_anten_* as equation S(1).. Where *E_ext_* is space electric field around this sensor (antenna) and *L_anten_* is the total length of antenna (see Figure S1a).

$V_{gap}\approx E_{gap}L_{gap}\approx L(structure,\varepsilon)E_{ext}$ S(1)

Where *L*(*structure*, *ε*) is the equivalent length of antenna considering the reduction of antenna gain caused by imperfect structure and the insulated packaging or filling materials with relative permittivity *ε*. *E_gap_* is the electric field intensity of central gap. It can be simulated or measured out.

As for the electrostatic force which equals to the electric field force applied on one electrode which is generated by the charges on the other electrode, equation S(2) is derived as:

$f\approx\frac{q_{gap}E_{gap}}{2}$, , $f=\frac{\varepsilon_{gap}\varepsilon_{0}S_{gap}E_{gap}^{2}}{2}$ S(2)

where *f* is the force applied on central beam (see Figure S1b) and *q_gap_* is the quantity of electric charge on central beam. *ε_gap_* is the relative permittivity of gap medium (air, *ε_gap_* =1) and *ε_0_* is the permittivity of free space, *S_gap_* is the approximative area of gap (near to the area of needle end face or central beam).

The force *f* causes the deformation of central beam as equation S(3) shows under static state:

$Dh_{cen}=f$ S(3)

Where *D* is stiffness factor of beam and *h_cen_* is the deflection of central beam. The back side of central beam section and fiber end face form a micro Fabry-Perot (F-P) interferometer. The relationship of light signal change Δ*I* of and *h_cen_* is given as equation S(4):

$\Delta I=\frac{k_{slope}\lambda}{L_{fp}}h_{cen}$ S(4)

Where *L_fp_* is the length of F-P cave and *λ* is the wavelength of light signal, and *k_slope_* is the slope of F-P reflection spectrum (see Figure S1c). So the relationship of gap electric field intensity *E_gap_* and light signal change Δ*I* is given as:

$\Delta I=\frac{k_{slope}\lambda S_{gap}\varepsilon_{gap}\varepsilon_{0}}{2L_{fp}D}E_{gap}^{2}$ S(5)

**
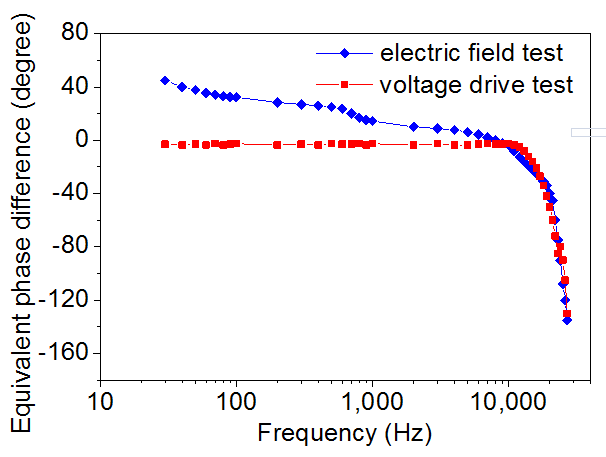
**

**Figure S2 |** **The equivalent phase difference (EPD).** EPD equals to **Δ***t_peak_* divided by *T_EF_* and multiply by 360 degree. Where **Δ***t_peak_* is the time difference between the peak of E-field waveform and the peak of light waveform, and *T_EF_* is the period of electric field.


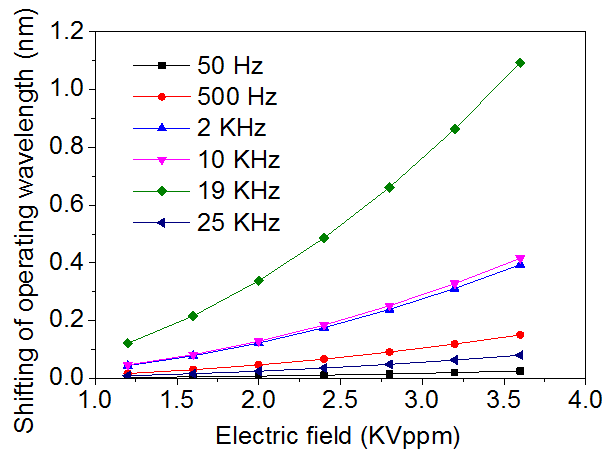


**Figure S3 | The shifting of operating wavelength.** It equals to the change of the mean value of light signal divided by spectrum slope *K_slope_*.
